# A survey to gather perspectives of DBT/Wellcome Trust India Alliance-funded researchers on public engagement with science

**DOI:** 10.12688/wellcomeopenres.17120.2

**Published:** 2022-04-08

**Authors:** Sarah Iqbal, Banya Kar

**Affiliations:** 1 Independent Communications and Public Engagement Consultant, Noida, 201304, India; 2 DBT/Wellcome Trust India Alliance, Hyderabad, India; 3 Freelance Science Communications and Public Engagement Professional, Noida, 201305, India

**Keywords:** Public Engagement, Science Communication, Researcher, India, Survey, Research, Biomedical science, Health research, Clinical research

## Abstract

Lately, the Indian research ecosystem has seen an upward trend in scientists showing interest in communicating their science and engaging with non-scientific audiences; however, the number and variety of science communication or public engagement activities undertaken formally by scientists remains low in the country. There could be many contributing factors for this trend. To explore this further, the science funding public charity in India, DBT/Wellcome Trust India Alliance (India Alliance), in a first of its kind of study by a funding agency in India, surveyed its 243 research grantees in November 2020 requesting their views on public engagement with science in India through an online survey. The survey included both quantitative as well as open-ended questions to assess the understanding of, participation in, and attitude of India Alliance Fellows/Grantees towards public engagement with research, identify the enablers, challenges, and barriers to public engagement for India Alliance Fellows/Grantees, understand the specific needs (training/capacity-building, funding, etc.) and develop recommendations for India Alliance as well as for the larger scientific ecosystem in the country. The survey showed that India Alliance grantees are largely motivated to engage with the public about science or their research but lack professional recognition and incentives, training and structural support to undertake public engagement activities.

## Introduction

It is being increasingly observed and accepted that advancements in science and technology towards improving human health and planetary well-being can be made more sustainable and equitable through building mutual understanding and collaboration between scientists and the public (defined here as non-expert audience including policymakers, research participants, and lay audience) ^1– 5^. For this reason, science along with citizen engagement is considered as an important mechanism for achieving Sustainable Development Goals (SDG 2030) ^6^ of the United Nations, which are 17 interlinked global goals designed to be a "blueprint to achieve a better and more sustainable future for all".

The Science Social Responsibility (SSR) ^3^ policy of the Department of Science and Technology (DST), Government of India, also underscores the importance of engaging with diverse stakeholders to maximise the impact of science on society. The consultations as part of the Science and Technology Innovation Policy (STIP 2020) draft ^4^ of the Government of India laid emphasis on involving the public in formulating STIP 2020 to make the policy-making process “decentralized, bottom-up, and inclusive” and reflective of public’s expectations from science and technology. The COVID-19 pandemic has also underscored the importance for the scientific contribute to research to mitigate the crisis but towards risk communication and public community, who generate and receive new scientific knowledge, to be better prepared not just to engagement. During such health crises, alleviation of public anxiety, research uptake, and prompt evidence-based actions would rely significantly on the engagement of experts with policymakers, media and the public at large ^7, 8^.

Public engagement with science includes intentional, open, and bidirectional interactions that give scientists and the public opportunities for mutual learning—through sharing and acquisition of knowledge while appreciating different perspectives and contexts ^9, 10^. Awareness about the cultural relevance of science and the importance of multiple perspectives in scientific processes is critical to effective public engagement initiatives ^5^. While public engagement activities can be multifarious ^11^— community engagement, science outreach, research uptake, citizen science, patient-involvement, participatory action research, participatory arts, policy advocacy, and so on—their main objective remains the same and that is to bring science and society closer together for their mutual benefit and towards achieving a common goal. It is about designing opportunities for the public and scientists to together explore the meaning and implications of research and also shape research agenda. Therefore, a critical objective of public engagement would be to align the intent of science with aspirations of the public and vice versa. Needless to say, engaging with the public is of strategic importance for funding and research organisations to ensure that their research and innovation are trustworthy and valued by their stakeholders and the public at large ^1– 4^.

DBT/Wellcome Trust India Alliance’s (henceforth referred to as India Alliance) core mandate, as a science funding public charity in India, is to invest in transformative ideas and supportive research ecosystems to advance discovery and innovation to improve health and well-being. This includes making the process and outputs of science accessible to everyone to enable and strengthen connections between science and society. In line with this mission, India Alliance provides funding and anchors various public engagement programmes that bring the scientific community and the public together to share, deliberate and collaborate on important matters of science, especially human health, which have implications for the society and the planet at large.

Over the years, India Alliance has also been conducting workshops to enable scientists to become effective communicators when writing for or speaking to their peers or the public. These unique workshops also help scientists appreciate the value and benefits of engaging with non-scientific audiences.

While post-independence India witnessed a strong wave of government- and scientist-led concerted efforts to promote public understanding of science ^12^, unfortunately the progress of these initiatives and their impact (both short- and long-term) is not well-documented. Lately, the Indian research ecosystem has seen an upward trend in scientists showing interest in communicating their science to non-scientific audiences; however, the number and variety of science communication or public engagement projects undertaken formally by scientists remains low in the country (authors’ observation). There could be many contributing factors for this trend ^13– 16^. To explore some of these factors and to be able to better support its grantees’ public engagement activities, India Alliance conducted an online survey in November 2020 for its grantees. The survey aimed to build a robust evidence set, which accurately reflected the views and needs of researchers funded by India Alliance and informed its approach to public engagement with science.

Therefore, this survey aimed to:

1. assess understanding of, participation in, and attitude of India Alliance Fellows/Grantees towards public engagement with science
2. identify the enablers, challenges, and barriers to public engagement for India Alliance Fellows/Grantees
3. understand the specific needs (training/capacity-building, funding, etc.) and develop recommendations for India Alliance as well as for the Indian scientific ecosystem.

This survey, while modest in its scope, is first-of-its-kind in the country and, therefore, serves as a good starting point for more in-depth investigation into the barriers and enablers to public engagement with science in India. We recognise that successful public engagement requires a systems approach that brings together diverse expertise ^5, 11^ and that researchers are one of the many drivers of public engagement with science. Therefore, it will be useful to expand the survey in future to include perspectives of diverse groups including researchers, science communication and public engagement practitioners, and other ecosystem stakeholders to build a robust data set necessary to inform a national framework aimed at bridging the gap between science and society.

## Methods

A shareable survey was designed using Microsoft Forms. The survey employed a mixed methods strategy and included 24 closed-ended questions (multiple choice and Likert scale) and nine qualitative questions, similar to open-ended questions. The questions were grouped under four heads: personal details; under of, participation in, and attitude towards public engagement; enablers, barriers and challenges to public engagement for researchers; and training and capacity-building in public engagement. The questions were modelled on a similar survey carried out by Wellcome in 2016 to gather views of researchers on public engagement in Asia and Africa ^13^. The full survey questionnaire is available as *Extended data*.

The link to access the survey was shared by email on 27 November 2020 with 243 Indian researchers in receipt of India Alliance fellowships or grants at the time of the survey (including few whose fellowships/grants were yet to be activated). Out of these, 137 (male – 78; female – 59; 56.4 % response rate) responded to the survey. Of these, 90 respondents were basic science researchers, 22 were clinical researchers, and 25 were public health researchers. A majority, i.e. 81% of the respondents indicated working as an independent researcher for more than 4 years (time post-PhD). About 19% of the survey respondents are early career researchers (1–4 years post-PhD), 25% mid career researchers (4–6 years post-PhD), and 47% senior researchers (6–15 years post PhD); 9% of the respondents indicated having worked more than 15 years post PhD. Furthermore, 58% of the respondents were based at research institutions, 18% at higher education institutions, and 17% at central, state and private universities; these organisations geographically represent around 31 cities of India (9 respondents chose to not reveal their host institution in the survey). Periodic reminders were sent by email to the fellows to complete the survey. Three fellows who received funding from India Alliance for their public engagement projects were contacted via email after the survey for their views (in 50–150 words) on the public engagement funding programme of India Alliance. They were informed that their input is intended to be included in a report of the survey results, and they can choose to either stay anonymous or be credited. The data was collected from 27 November 2020 until 28 April 2021. Of the 106 researchers who chose not to respond to the survey, majority belonged to the intermediate career fellowship category of India Alliance.

Following the end of the survey, the data was automatically mapped in Microsoft Excel, a feature of Microsoft Forms, and was cleaned for duplication and errors and anonymized. Microsoft Excel tools were used to analyse and visualise the data.

The study did not undergo a formal ethical clearance process as the primary objective of the study was to gather insights of grantees to inform India Alliance’s public engagement support mechanisms. At the start of the survey, the objectives of the survey were clearly stated and the respondents were informed that the anonymized data of the survey may be communicated in the future. Participation in the survey was entirely voluntary with fields seeking personal information being non-mandatory; and, therefore, completion of the survey was taken as consent to participate.

## Results

### 1. Understanding of and participation in public engagement

***a) Understanding of public engagement***

**Figure 1.**
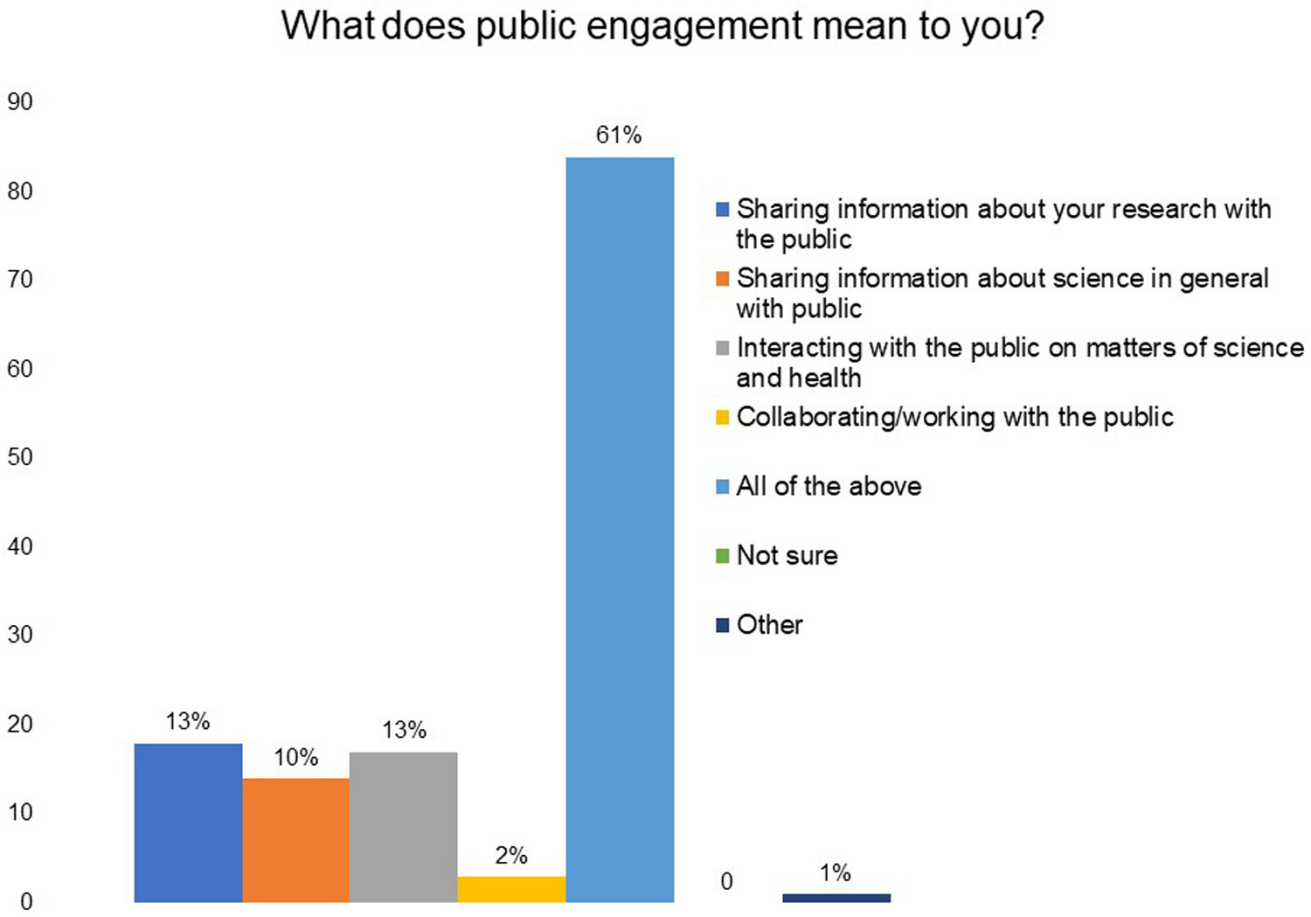
What does public engagement mean to you? 61% of the respondents said that public engagement to them meant sharing information about your research and science with the public, interacting with the public on matters of science and health, and collaborating with the public.

In Figure 1, it can be seen that in a multiple-choice question 13% of the respondents indicated that public engagement to them meant ‘sharing information about your research’, 13% indicated ‘interacting with the public on matters of science and health’, 10% selected ‘sharing information about science in general with the public’, 2% selected ‘collaborating/working with the public’ and 61% of the respondents said public engagement to them meant ‘all of these’.

A select few responses to the open-ended question on respondents’ understanding of public engagement is provided below:

*“Public engagement to me means not only interacting with public with matters of health, but also collaborating with them and understanding their needs and prioritizing research based on the community’s necessity. Every community is empowered enough to voice out their concerns if listened to, public health research should prioritize the concern of lay people, and as public health researcher(s), the best way to engage is to orient our research to provide solution (to) these concerns within the research interest/experience/capacity.”*
*“The questions that we seek to answer in our institutes/labs should arise in the field i.e., out of an extensive interaction, understanding, assimilation and conceptualization of a problem that is pertinent to the public, be it a public health problem or otherwise.”*
*“I think public engagement is one of the great ways to take science amongst the public. This also helps the researchers to express their research in simple language and take it beyond the scientific community and share the knowledge.*
*“Creating a dialogue with the public to make them understand ways of science and in turn understand their expectations.”*
*“Science and Society are dependent on each other. Science drives society and society drives science. So, the communication between them is indispensable.”*

***b) Perceptions of India Alliance grantees on public’s interest and understanding of science***

To gauge the perception of India Alliance grantees on public’s understanding of science, they were asked to rate the level of public understanding of science and health issues in India in a multiple-choice question. A total of 86% of the respondents believed that this understanding of science and health issues is low while 5% felt it was high and 9% were not sure. Ninety seven percent of the respondents believe that the public in India is interested in learning more about science. . Out of these, 38% of the respondents feel that while the public is interested, scientific information is not readily available to them, 23% believe that the public is interested but lacks understanding of science and technology, and 15% indicated that the public is only interested in health, food and applied sciences.(see extended data).

***c) Participation in public engagement***

**Figure 2.**
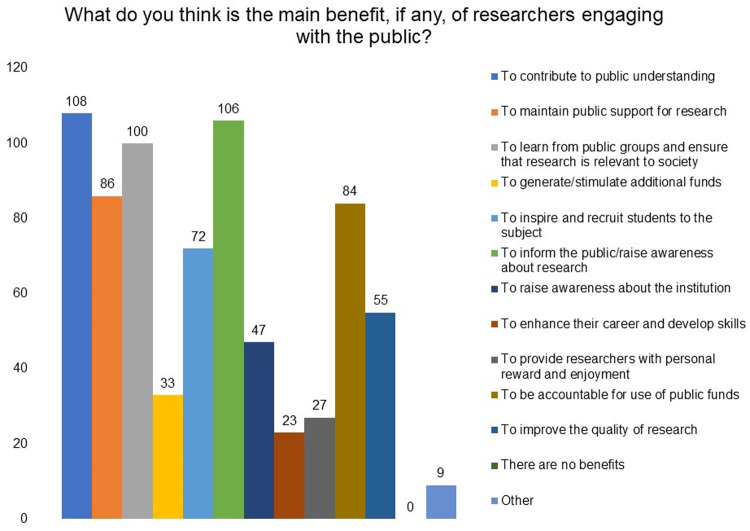
What do you think is the main benefit, if any, of researchers engaging with the public? Respondents were asked to choose all options that apply and ‘contribute to public understanding of science’ emerged as the top benefit.

**Figure 3.**
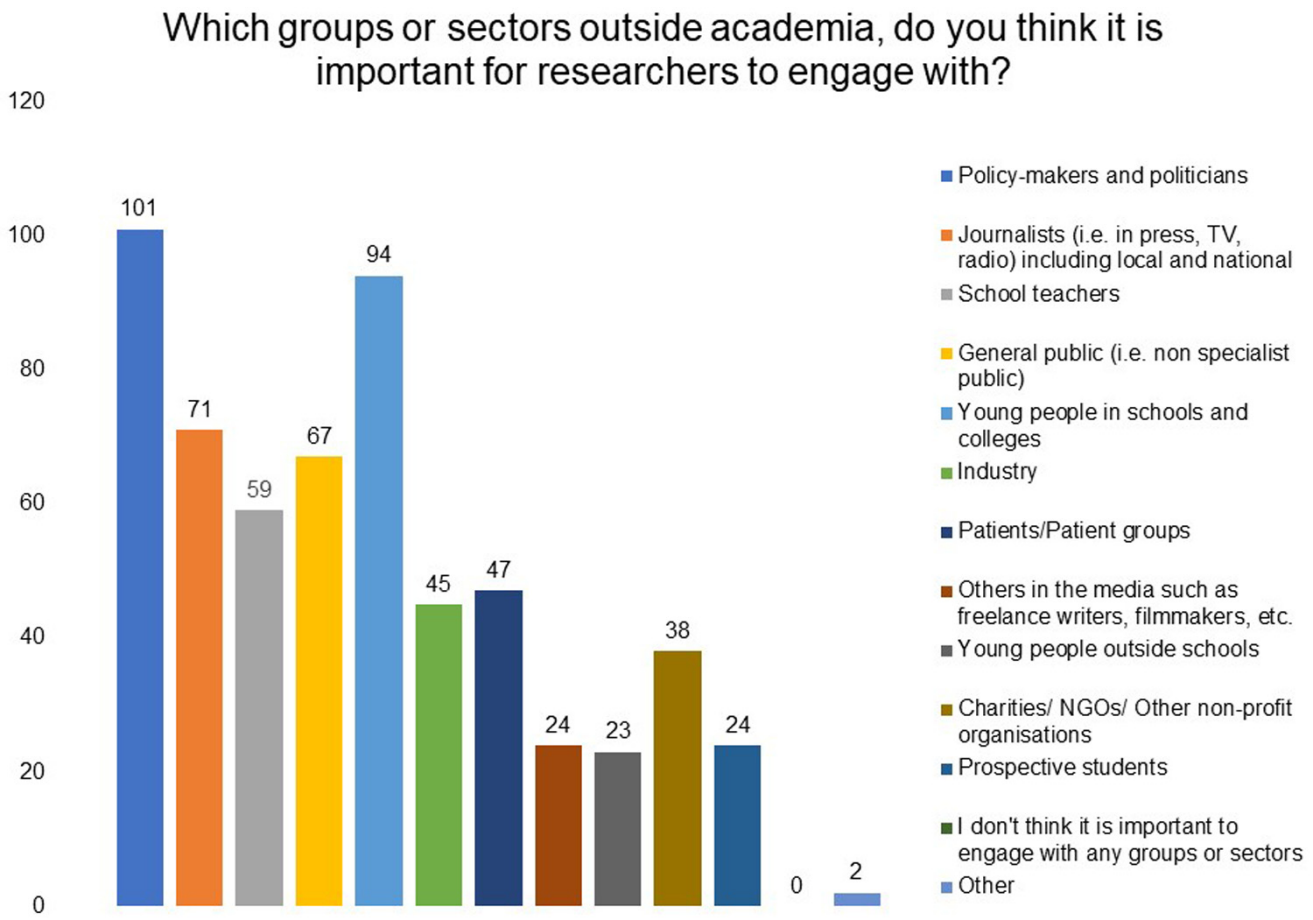
Which groups or sectors outside academia do you think it is important for researchers to engage with? Respondents were asked to choose three options,which were the most important for their research or for biomedical and health research in general, from the list provided. Policymakers and politicians, young people in schools, and media/journalists were indicated as the three main groups.

A majority of the survey respondents (88% where 52% strongly agreed and 36% agreed) were in agreement with the statement that engaging with the public on matters of science and health is the responsibility of the researchers (see extended data). Respondents selected ‘contribute to public understanding of science’, ‘inform the public/raise awareness about research’, and ‘learn from public groups and ensure that research is relevant to society’ as the top three main benefits of engaging with the public ( Figure 2). Policymakers and politicians, young people in schools and media/journalists were indicated as the three main groups they would like to engage with ( Figure 3).

**Figure 4.**
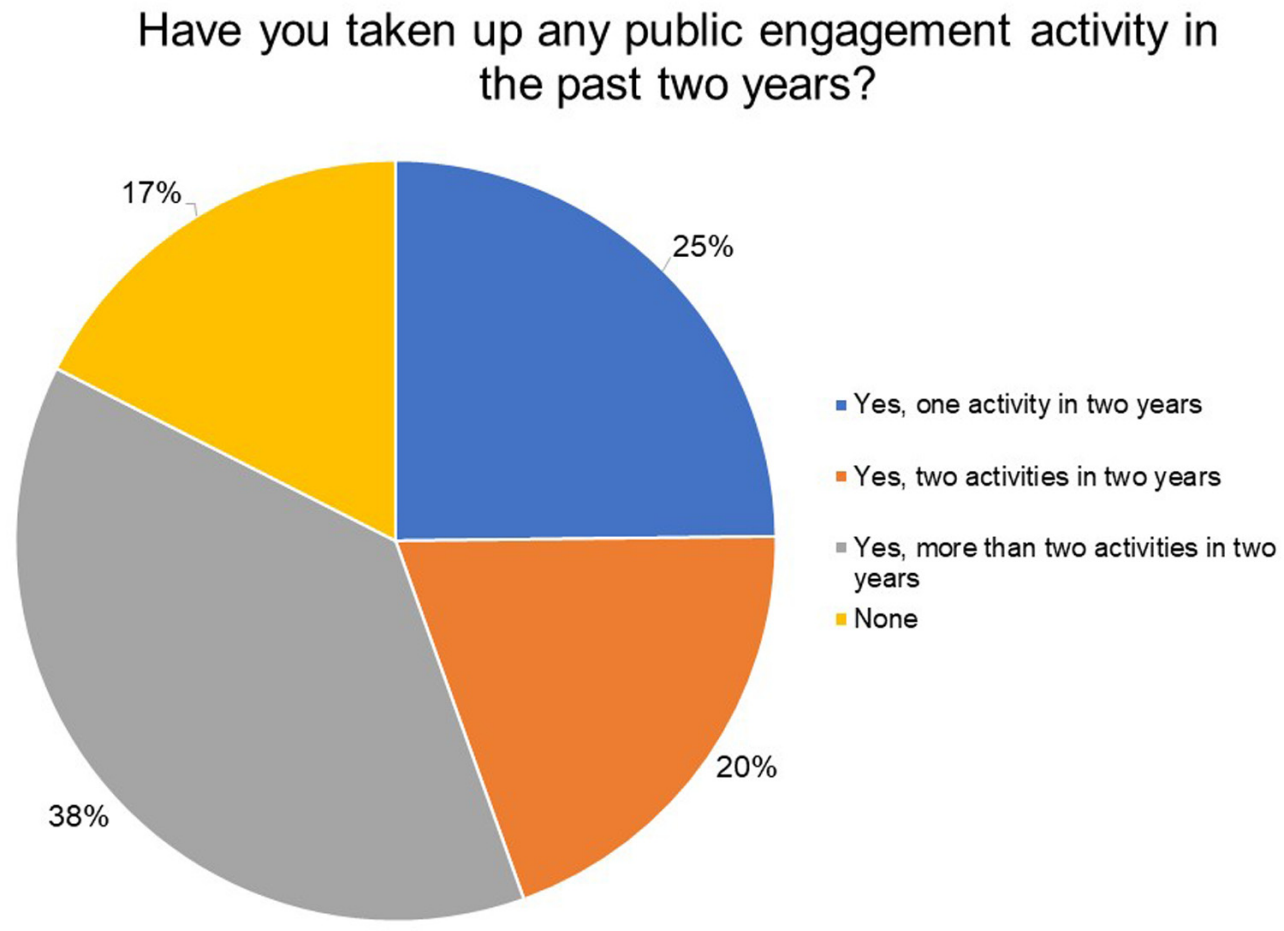
Have you taken up any public engagement activity (or shared your science with the public at large) in the past two years? 83% of the respondents participated or undertook at least one public engagement activity in the last two years.

To get a sense of how often our grantees engaged with the public, we asked them if they had taken up any public engagement activity or shared their science with the public at large in the past two years. From Figure 4, 83% of the respondents participated or undertook at least one public engagement activity in the last two years—25% undertook one activity, 20% undertook two activities and 38% respondents undertook more than two activities in two years. Out of these, 52% of the respondents said they would like to spend more time engaging with the public. In contrast 42% said they were content with the time they are currently spending on public engagement activities and 4% are unsure about how much time they would like to devote to this.

Out of the 17% who undertook no activities in the last two years, 80% did not receive any opportunity to engage with the public and 20% received an opportunity but did not participate. Out of this, respondents who had the opportunity but did not participate, 60% believed engaging with the public on matters of science and health is the responsibility of the researchers, whereas 40% were neutral. Furthermore, out of these who did not participate in any public engagement activities, 58% of the respondents said they would like to spend more time, 12% said they were content with time they are currently spending on these activities and 30% said they were not sure how much time they would like to devote to this.

***d) Nature and scope of public engagement activities***

**Figure 5.**
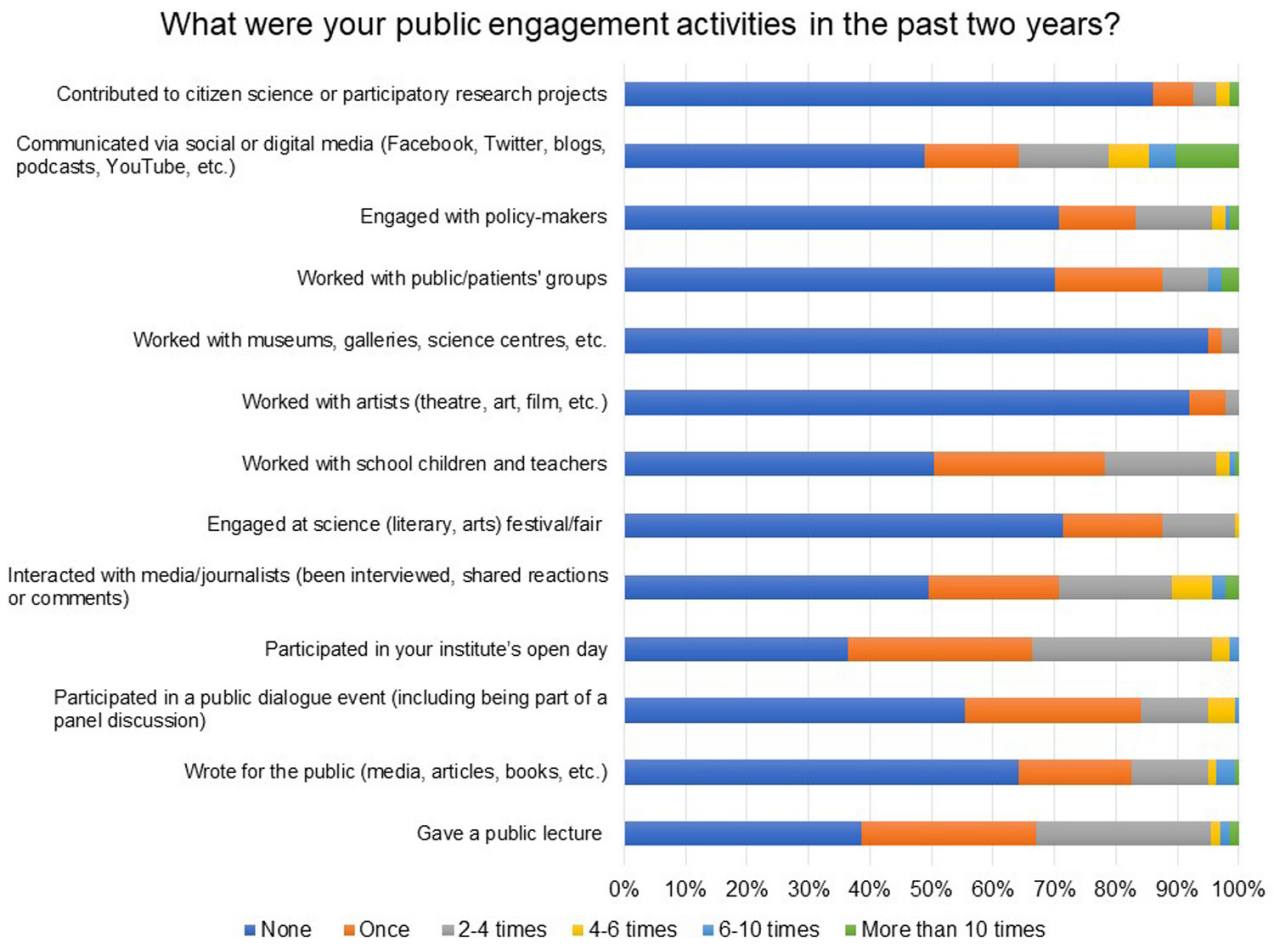
What were your public engagement activities in the past two years? Respondents were provided a list of activities and were asked to indicate the number of times (None, Once, 2–4 times, 4–6 times, 6–10 times, More than 10 times) they had undertaken the activity.

In a Likert scale question, respondents were provided a list of activities and were asked to indicate the number of times (None, Once, 2–4 times, 4–6 times, 6–10 times, More than 10 times) they had undertaken the activity. Public lectures, panel discussions, open day events at institutions, writing media articles and social or digital media (Facebook, Twitter, blogs, podcasts, YouTube, etc.) appear to be popular modes of communication and engagement used by the respondents ( Figure 5). In total, 59% of the respondents pursuing clinical and public health research indicated working with public/patient groups.

Only 5% of the respondents indicated that they worked with artists, museums, galleries, science centres, etc. and contributed to citizen science or participatory research projects. A few of them shared details of their ongoing public engagement projects (see *Underlying data*). 

### 2. Enablers, challenges, and barriers to public engagement for researchers

***a) Institutional support***

Through this survey we wanted to understand the support at organization level for public engagement. When asked to choose and indicate if their respective organizations were supportive (or not supportive) of public engagement activities, and 89% of the respondents indicated that their institutes/organizations were either fairly (50%) or very supportive (39%) of public engagement activities. A total of 10% of the respondents chose to remain neutral (see extended data). The exact nature of this support offered by institutions, whether in spirit or tangible, was not ascertained through this report.

89% of the respondents indicated that their institutes/organizations were either fairly or very supportive.

***b) Challenges to public engagement***

**Figure 6.**
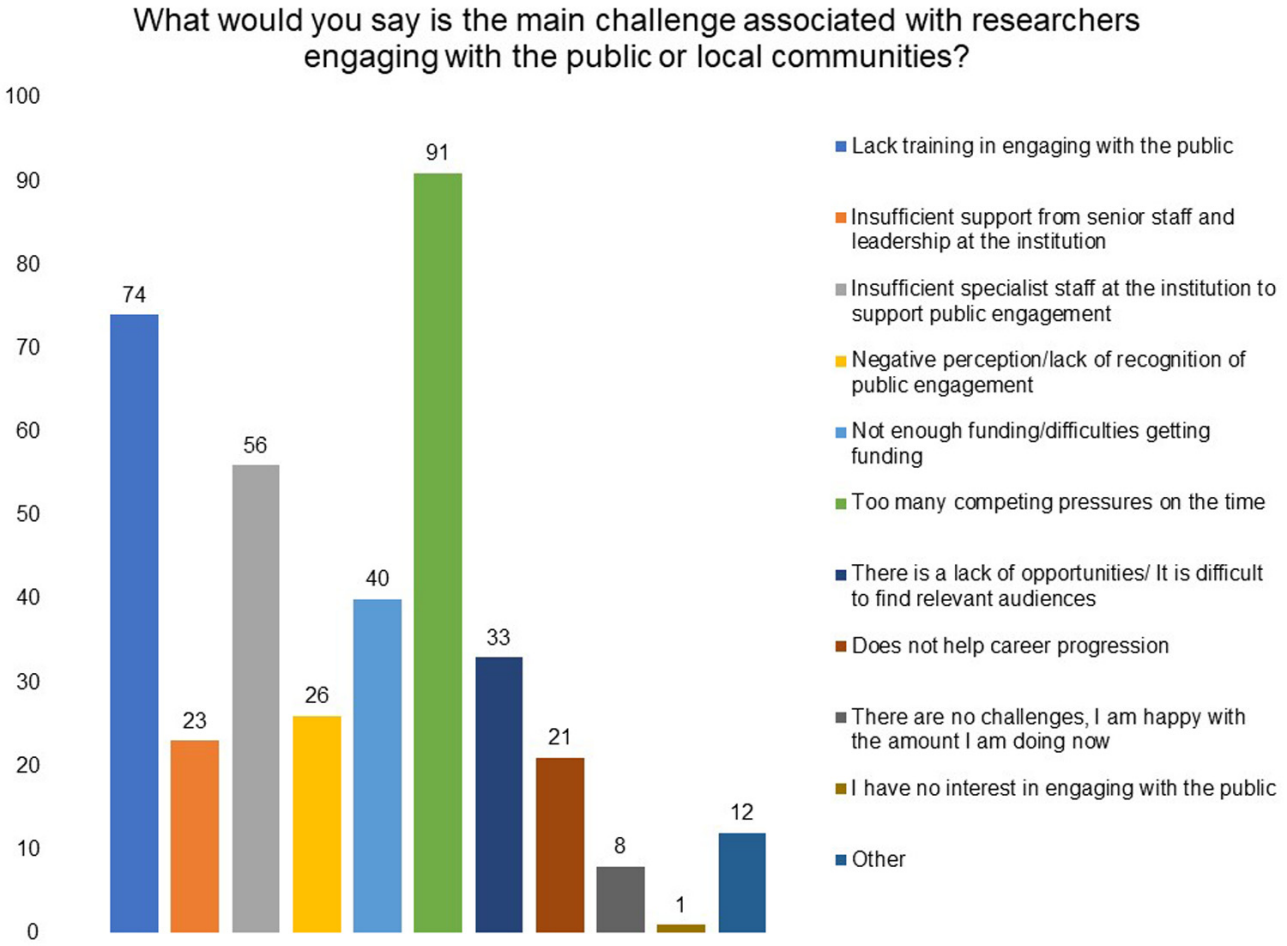
What would you say is the main challenge associated with researchers engaging with the public or local communities? Respondents were asked to choose three most important options in their opinion from a list.

To understand the main challenge associated with researchers engaging with the public or local communities, we asked the respondents to choose the three most important options, in their opinion, from a list. ‘Too many competing pressures on time’, ‘lack of training in engaging with the public’ and ‘insufficient specialist staff at the institution to support public engagement’ emerged as the top three challenges ( Figure 6).

After the above-mentioned factors, ‘not enough funding/difficulties in getting funding’ and lack of opportunities/difficult to find relevant audience’ also stand out as challenges to public engagement.

***c) Enablers for public engagement***

In a Likert scale question where respondents were asked to rate importance of a list on a scale of 1 to 5 (where 1 is not important and 5 is very important), ‘if my institution had a public engagement specialist’ (58% rated 5 & 4), ‘if it was easier for me to get funds for engagement activities ’ (54% rated 5 & 4) and ‘if my public engagement work was recognised and valued more’ (49% rated 5 & 4) stood out as the most important factors that would encourage researchers to get more involved in engagement with the public or local communities. Training in public engagement, more support from public engagement specialists at institutions, and time for public engagement activities also stood out as important enablers (see extended data).

The observations with regards to enablers, which can encourage researchers to take up public engagement projects, correlates with the indication that competing pressures on time and lack of public engagement expertise are the two major challenges to be addressed to increase chances of researchers taking their science to the public.

Below are inputs shared by some of the respondents in an open-ended question:

*“The senior leadership always encourages public engagement, however competing interests from other institutional and research duties make this relatively less a priority. Also, the lack of proper training makes me feel less confident.”*
*“As researchers, we lack training on how to engage with the public and simplify our science to reach the right audience. Public engagement specialists do not exist in our kind.”*
*“Writing media articles and engaging with the press is easy. Getting a physical audience of patient groups is a challenge.”*

In a Likert scale question of scale of 1 to 5 (where 1 is not important and 5 is very important), respondents were asked to rate a list on how they feel the support for public engagement or the delivery of public engagement by researchers could be improved in India. ‘Increase recognition associated with public engagement work’ (81% rated 5 & 4), ‘Increase or provide dedicated public engagement staff at institutions’ (80% rated 5 & 4) and ‘Communicate importance of public engagement’ (76% rated 5 & 4) emerged as the three top factors to improve support for or delivery of public engagement (see extended data). The other options on the list were—increase training for public engagement, increase funding for public engagement, embed public engagement in main research grant, and provide dedicated time for public engagement at institutions. The results again mirrored the barriers and incentives highlighted in earlier questions.

Following are some of the thoughts shared by the respondents in an open text optional question:

*“Provide dedicated time for public engagement at institutions—oftentimes, most researchers, particularly like mine, are engaged with multiple meetings, deadlines, grants, reviews and classes. If a granting agency or institute mandates that a certain number of hours every month or year should be spent on public engagement, then there is a good chance that most researchers would 'make' time for it.”*
*“Support from the local and central government for public engagement with researchers is critical specially for not-for-profit research organizations. Though our organization has been working in these areas for 20 years, with evolving situations the main challenge sometimes is to get an approval from the local health authorities before one can talk to the grassroot level workers and local medical staff.”*
*“Researchers need more training on this because they are not exposed to what happens in the field. Online courses/seminars by leading international researchers from diverse backgrounds on public engagement may be introduced and circulated in all institutions. Social scientists must be pulled up in this. Institutes need to be engaged at the top level. The outreach and advocacy activities must be given points to researchers during their promotions.”*
*“Public engagement is not recognised as part of research activity. Thank you for bringing this into limelight. I am sure it will change for the better.”*
*“Public engagement is a new concept. It is strongly promoted by funding agencies from high-income countries. Most funding agencies in the Indian context are yet to recognise its need and allocate parts of their budget for such an activity. It is important for researchers to be sensitised to public engagement, its importance, and its objectives. Making public engagement a necessity within funding applications is therefore important to promote the concept. However, to do so, funding agencies themselves need to be sensitised to the concept.”*

India Alliance offers funding for public engagement projects that propose to develop novel and creative methods and tools to empower researchers to communicate and engage with the public effectively ^17^.

A basic biomedical research fellow of India Alliance, who also received the India Alliance’s Public Engagement (PE) award, stated, *"The Public Engagement Funding of DBT/Wellcome Trust India Alliance is a one-of-a-kind opportunity to engage in science communication. There are several modes of science communication including but not limited to science columns, public speeches, fine and performance arts. The Public Engagement Award allows the fellow to pick any media of science communication and effectively engage with the non-scientific community. The IA has a deep commitment towards science communication and has a dedicated team for such activities, which allows the fellows to explore and design tailor-made public engagement activities. As an IA fellow who has availed this funding, I strongly urge others to explore the Public Engagement Funding opportunity, which will be an enriching and rewarding experience."*

Another India Alliance fellow based at a medical institution had this to say about their experience of utilising India Alliance’s funding for public engagement *“Firstly, IA’s PE initiative is unique and I believe enables IA fellows to go beyond research and connect with communities and stakeholders that they engage at a level that may not have been otherwise possible. It was fulfilling, as we came face to face with the creativity and emotions of the people that we engaged with, as we broke barriers to connect with them on a very different plane. Secondly, our workshop on Comics for TB brought together healthcare workers including doctors, helping them disconnect from their routine. We saw their enthusiasm grow through the course as evidenced by the quality of their comics and the stories they developed. Thirdly, at a personal level, the workshop has opened doors to a whole new prospect, i.e., of communicating medicine through art, which I hope to pursue in the future in whatever way possible. Overall, it was a very fulfilling experience and I am honored to have received the grant and the experience it brought thereof.”*

We asked the respondents in an open-text question, if they had applied for grants for their public engagement activities to any funding agency/organisation other than India Alliance in the country. Some respondents had approached the following funding sources for public engagement: Wellcome Trust, UK; Ministry of Ayurveda, Yoga & Naturopathy, Unani, Siddha and Homoeopathy (AYUSH), Government of India; Dr Ramachandra N Moorthy Foundation for Mental Health and Neurological Sciences, National Institute of Mental Health & Neurosciences (NIMHANS); Science and Engineering Research Board, Department of Science and Technology, Government of India (DST SERB grants mandate one public engagement activity per year for its funded grants and provides specific additional funding for that purpose); IndiaBioscience Outreach Grant; Department of Biotechnology (DBT), Government of India; Indian Council of Medical Research (ICMR), Government of India; Indian Council of Social Science Research (ICSSR), Ministry of Education, Government of India; Indian National Young Academy of Science, Indian National Science Academy (INSA-INYAS); philanthropic foundations; non-profit organizations like Campaign for Tobacco Free Kids, World Diabetes Foundation, and Encephalitis Society; and intramural institutional funds.

***d) Training and skills for public engagement***

**Figure 7.**
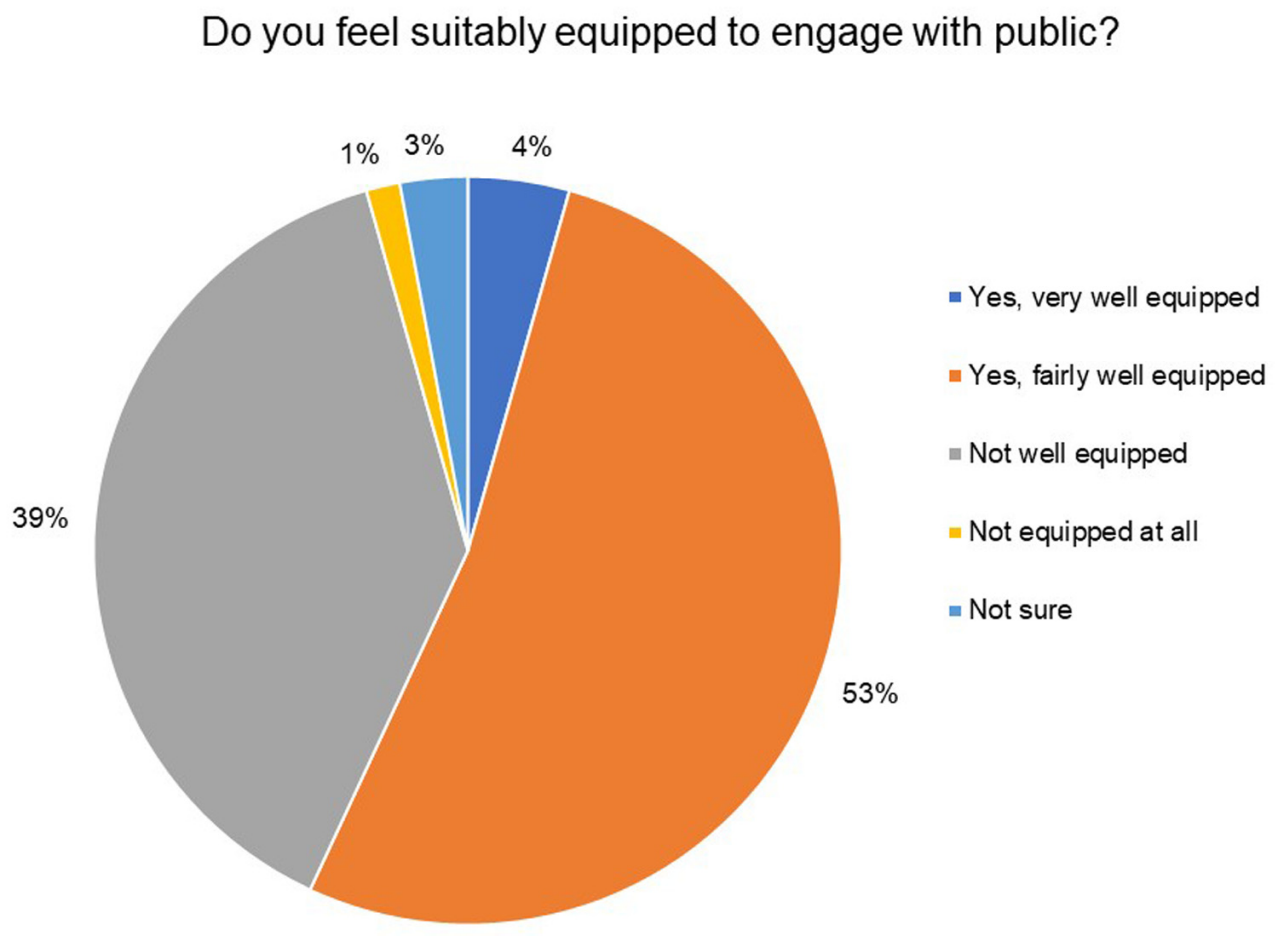
Do you feel suitably equipped to engage with the public? 57% of the respondents felt fairly or very well equipped to engage with the public.

Through multiple choice questions, we found that while 57% of the respondents felt fairly or very well equipped to engage with the public, as low as 3% and 10% of the respondents had formal or on-the-job training in public engagement, respectively ( Figure 7).

Notably, a large percentage of the respondents did not have the opportunity to train in public engagement. 89% of the respondents indicated the absence of any formal training opportunities in public engagement at their institution and 80% of the respondents did not receive any formal public engagement training (see extended data).

The respondents were asked what kind of training they would require to be able to engage better with the public. This was a Likert scale and respondents had to rate a list on a scale of 1 to 5, where 1 is not important and 5 is very important. To better engage with the public, the respondent chose the following as the top three areas of training (see extended data): how to organise/run a public engagement activity (72% rated 5 & 4), engagement with schools/children/young people (69% rated 5 & 4), and engagement with policy (68% rated 5 & 4). The other options enlisted were—public engagement in general/introduction to public engagement, public engagement tools and methodologies, media training on being interviewed by journalists, writing for the public, speaking for the public, using social media/digital media, preparing project proposal for public engagement funding, and writing Pathways to Impact statements.

### Summary of survey results

Our survey suggests that the surveyed India Alliance grantees demonstrate a clear interest and motivation for public engagement; the majority of them agree that as researchers they play an important role in facilitating public's access to science. While the respondents agree that public understanding of science and health issues in India is low, they believe that the public is interested in science but does not have access to scientific information and may not understand science very well to begin with. Although 83% of respondents indicated that they undertook at least one public engagement activity in the last two years, more than half of the respondents also said they would like to spend more time engaging with the public. Furthermore, it was encouraging to note that the institutions of the majority of the respondents were generally supportive of their public engagement activities. However, the nature of this support is unknown.

‘Contribute to public understanding of science’, ‘inform the public/raise awareness about research’, and ‘learn from public groups and ensure that research is relevant to society’ were identified as the top three benefits of engaging with the public in the survey. Respondents also indicated other benefits: to build public’s trust and support for scientific research; to enhance their career and develop skills; to garner feedback from different perspectives and areas of expertise for improvement of research; to get more funds and resources for research through public support; to motivate young people to pursue a career in research; to find collaborators for work; to fight misinformation; and so on.

For 61% of the respondents, public engagement means not only sharing their research or scientific information with the public, but it involves interaction and collaboration with them on matters of science and health. This view was supplemented by the response provided by the researchers to the open-ended question where we asked them to elaborate their understanding of public engagement. However, the survey data shows that grantees mostly undertook activities that involved one-way communication such as giving public lectures, writing media articles and social or digital media (Facebook, Twitter, blogs, podcasts, YouTube, etc.). This could largely be due to the lack of exposure to the value or tools and methods of two-way communication and engagement. The data for India in the survey run by Wellcome in 2016 also supports this observation “... *the public engagement culture in India seemed more to be reaching out to school children and education, often to encourage future funding back to the institution or research lab or because government quotas encourage this” ^13^.*

Respondents considered policymakers and politicians, young people in schools and colleges and journalists (i.e., in press, TV, radio) as important groups or sectors outside academia to engage with. It was encouraging to note that grantees believe that learning from public groups and ensuring that research is relevant to society is an important benefit of public engagement in addition to improving public's understanding of scientific research and its impact.

The majority of the respondents indicated competing pressures on time, lack of training in engaging with public and insufficient specialist staff at the institution to support public engagement programmes were the major challenges. In line with the identified challenges, the respondents identified institutionalization of public engagement support through funding and dedicated personnel along with formal recognition for public engagement work as major enablers to boost engagement of researchers with the public. With the majority of the respondents (88%) having no access to any formal public engagement training facilities at their institutions, interventions for capacity building in public engagement at institution level emerges as a major gap to be addressed in the Indian science ecosystem. See Table 1 for a summary of the major findings from the survey. 

**Table 1.** Major findings about public engagement activities of respondents; challenges, barriers, and enablers for public engagement by researchers; and training requirements of researchers to undertake public engagement activities.

Top three main benefits of researchers engaging with the public	To contribute to public understanding of science To inform the public/raise awareness about research To learn from public groups and ensure that research is relevant to society
Top three groups or sectors outside academia, most important for researchers to engage with	Policymakers and politicians Young people in schools Media/journalists
Most popular modes of communication and engagement activities used by the respondents of the survey	Public lectures, panel discussions, open day events at institutions, writing media articles, and social or digital media
Main challenges associated with researchers engaging with the public or local communities	Too many competing pressures on time Lack of training in engaging with the public Insufficient specialist staff at the institution to support public engagement
Top three factors that would encourage researchers to get more involved in activities to engage the public or local communities	If my institution had a public engagement specialist If it was easier for me to get funds for engagement activities If my public engagement work was recognised and valued more
Top three factors to improve support for or delivery of public engagement activities by researchers in India	Increase recognition associated with public engagement work Increase or provide dedicated public engagement staff at institutions Communicate importance of public engagement
Areas of training researchers in India would require to be able to engage better with public	How to organise/run a public engagement activity Engagement with schools/children/young people Engagement with policy

## Discussion and recommendations

The survey reported here focused on understanding the needs of researchers funded by India Alliance, who represent basic biomedical, clinical and public health researchers based at various academic and research institutions in India, so that appropriate support can be provided to them to undertake public engagement activities as part of their fellowship or grant programme. Despite the fact that the sample size of Indian researchers in the Wellcome Trust survey conducted in 2016 was relatively small (n=18), it was interesting to see the similarity in challenges and barriers to public engagement identified by the respondents in the two surveys, indicating that little progress has been made on this front since the Wellcome survey.

Since the respondents come from different fields of research, factors influencing their understanding of and participation in public engagement may vary. For example, a public health or a clinical researcher may receive more opportunities to engage with non-science audiences compared to a basic biomedical researcher. Therefore, the former may view engagement with the public as an important part of their research programme and consequently attach more value to it. Similarly, interest in public engagement may also vary depending on the career stage of a researcher. Considering this heterogeneity and that of institutional cultures in the country, further studies can look at context-specific factors, including their career stage (early career versus senior researcher), influencing researchers' motivation and ability to engage with public groups. Funding agencies and institutions can undertake such assessments on a periodic basis to respond more proactively to the evolving needs of researchers’ and that of society. Additionally, it might be useful to address the non-response bias in this study that may possibly generate new insights that can be utilised by India Alliance or other funding organisations to design a public engagement support programme for its grantees. It is possible that the insights generated through this survey represent views of researchers who are largely interested in public engagement.

These variations in the responses notwithstanding, since the surveyed researchers are based at institutions in different Indian cities, their views can be considered as a reasonable representation of the biomedical and health research community in the country. Consequently, the recommendations based on the survey responses presented here are broadly applicable and can serve as possible interventions that funding agencies and academic and research institutions in India can implement to enable public engagement efforts of these researchers.

### 1. Training and building capacity for public engagement

*“Public engagement should be part of grooming researchers and should commence early. Instead of placing the responsibility on the researchers (who are often hard-pressed for time) to educate themselves on the matter, it would be desirable if the Host Institute/Funding agency facilitates a mandatory Public Engagement Training module that includes public engagement assignments. A structured approach will help us (researchers) understand the process and practice it.”* India Alliance fellow
*“Formal training to scientists like me in public engagement and additional funds and dedicated support staff would be of great help to organize public engagement activities outside my time for routine lab-based science. I feel it is important for the public to know and understand why we are spending so much money on our research projects and to know how the outcome of our research would benefit society.”* India Alliance fellow

Access to tools and resources along with training in public engagement were cited as critical challenges due to which researchers do not feel sufficiently equipped to engage with the public, particularly with certain public groups such as the media/journalists and policymakers. While clinical and public health researchers get to work with patient and public groups as part of their research, basic science researchers working in laboratory settings generally lack this exposure.

To address this training gap, PhD programmes can include training modules on public engagement with science. Regular courses and hands-on workshops, with case studies from India, can be organised for students and researchers to help them appreciate and integrate public engagement practice in or alongside their research programmes. In these training courses, special care needs to be given to convey the difference in communication style and engagement strategy depending on the objective (awareness-raising, consultation, collaboration, etc.) and audience (for e.g., school student, a journalist, policymakers, community group, etc.).

Examples of such courses, training and fellowships for researchers are University of Cambridge Engaged Researcher training online edition, British Science Association, British Science Association Media Fellowships, Royal Society Pairing Scheme

*“Public engagement requires an ongoing commitment and effort, and it's vital to find time for the same as it involves several deliberations with important stakeholders to make it successful. A separate wing of professionals are required to organise and execute the public engagement initiatives.”* India Alliance fellow

In addition to providing training to students and researchers, it will be equally important to develop a cadre of professionals who can design and deliver public engagement programmes with and alongside the researchers and act as critical drivers of research and practice in public engagement in India. These professionals can serve as important connectors between science and society. Over time, specialised training courses can be designed and delivered to build this professional capacity in a more systematic and needs-based manner to support and sustain public’s engagement and involvement in science in India. Examples of such courses are MSc in Science Communication and Public Engagement offered by University of Edinburgh, UK; MSc Science Media Production, Imperial College London, UK, etc.

### 2. Incentivise public engagement

*“I think public engagement for researchers becomes challenging due to lack of time and lack of help that is required to conduct an event to engage with the public. With some help offered from the institute and some help from a funding agency, it is possible for researchers to do this more often and more comfortably.”* India Alliance fellow
*“It would really benefit if the efforts made by the researchers towards public engagement activities are recognized, appreciated and encouraged by respective institutes.”* India Alliance fellow

Owing to competing pressures on their time, particularly during early years of an independent research career, researchers find it challenging to undertake or participate in public engagement activities. These challenges were highlighted not just by our survey respondents but by a diverse group of researchers during a listening session organised by India Alliance and the U.S. Department of Health and Human Services (HHS) Office of Global Affairs (OGA) on Fostering International Research Cooperation – Enabling Mobility, Research, and Capacity Building in 2019 ^18^.

While institutions and funders in India are largely supportive of public engagement activities, these efforts are not a criterion for tenure, promotion, or funding. Additionally, researchers are not expected to formally allocate time for these activities as they would for other institutional and academic responsibilities. This further diminishes the enthusiasm and importance of public engagement in view of other responsibilities such as teaching, administrative and editorial roles that are considered for professional advancement. Dedicated time for public engagement, particularly if it is not already part of a researcher's academic programme, and due weightage wherever appropriate, should be given to public engagement projects/initiatives in funding proposals and faculty recruitment and promotions.

*“Public engagement is very important but due to several other scientific and academic responsibilities, we (researchers) are not able to give enough time to it. Probably, it would be a good idea to include public engagement activity as a part of scientific project proposals and provide some additional funds to carry out such public engagement activity.”* India Alliance fellow

Respondents of this survey also cited lack of funding for public engagement as a critical barrier. Development of an impactful public engagement programme is a time and resource intensive process. It requires dedicated personnel and multidisciplinary teams. More tangible support from the institution in the form of funding or dedicated communication, public or community engagement specialists, would enable researchers to build meaningful and sustainable connections with the public group(s) of their interest.

*“Integration of public engagement with funding mechanisms will ensure that it is taken up seriously in the beginning and later, it may become natural and more active participation might come up. Importantly, institutions should appoint a public engagement officer to help with the content development for public engagement to make it more appealing and engaging for the public.”* India Alliance fellow

For public engagement activities to have a real-world impact, they need to be carried out in a purposeful, sustained and evidence-based manner. Therefore, plans to engage with the public and other stakeholders should be integrated in research projects right at the start and not appear as an after-thought. This would be particularly critical for research programmes that rely on research uptake, community or public participation or policy engagement to be impactful. This would also ensure that public engagement plans support research goals and do not serve as a distraction.

Funding agencies could consider, as appropriate, providing ring-fenced funds in a research project towards costs related to public engagement activities which could also include hiring of communication or public engagement specialists. For example, India Alliance’s Team Science and Clinical and Public Health Research Centre grants provide funds for research management to support and facilitate multi-centre collaborations, a key attribute of these grants. The global science funding charity, Wellcome, provides Research Enrichment Funding to its grant holders to help them improve the impact of their work. In a similar vein, DELTAS programme of the African Academy of Sciences provides seed grants to its doctoral and postdoctoral trainees to undertake innovative community and public engagement projects aligned to their research projects with the dual objective of building skills and capacity for public engagement and to change mindsets.

Increasingly, various international research grants such as UK Research and Innovation (UKRI), The Global Fund, National Institute for Health Research (NIHR) UK, etc., require applicants to share a 'pathway to impact’ that outlines how they plan to make the stakeholders and/or beneficiaries aware of their research to achieve impact. Some of these international funders also require a plan for 'public or patient involvement’ in the design, conduct, and dissemination of health research.

Integration of public engagement in research proposals could possibly encourage researchers and their institutions to give sufficient thought and allocate resources and time to carry out public engagement activities and see value in it as well.

Funding agencies, institutions and other science foundations in the country could also consider instituting independent grants to promote public engagement with science. These would be particularly useful for researchers who do not have provisions in their research grants to undertake public engagement activities.

### 3. Build a culture of public engagement

The respondents cited recognition for public engagement work of researchers and the need to communicate its value and importance as critical enablers for public engagement with science in India. Accomplishing this would require a change in research culture and shift in mindsets. Therefore, in addition to tangible interventions (training, capacity building and funding), it will be important to build a culture of public engagement with science in India. Among other advantages, this would hopefully result in the scientific community not feeling burdened by the need to engage with the public but instead appreciate its importance and recognise it as part of their research practice. Also presently, scientists based at institutions that don’t support public engagement may find themselves at a disadvantage compared to their colleagues working in more enabling ecosystems. Creating a research culture in India that is supportive of such activities would create a level-playing field and ensure equal access to the benefits of public engagement to all researchers.

To this end, building a research culture that has communication and engagement with the public at its very core will be critical. This, to start with, will require policy-level interventions with a shared understanding of the research community that while uptake of research knowledge and new technologies requires sustained engagement with the public, this engagement also informs and makes research ethical, socially relevant and useful. With this in mind, funding agencies and research organisations can include public engagement in their core mandate and overall institutional strategic framework.

More fundamentally, to enable such a culture, organisations and individuals involved in research would need to build a common understanding of the role public engagement plays in shaping research and its potential impact on human health and planetary well-being. Building such a culture would also enable the members of the public to recognise and appreciate their role in science. See Table 2 for a summary of these recommendations. 

**Table 2.** Summary of Recommendations

Training and building capacity for public engagement	Develop specialised courses and training workshops for researchers, students and public engagement professionals
Incentivise public engagement	Integration of public engagement in research proposals Include public engagement activity as part of evaluation for faculty recruitment and promotion Institutions can hire dedicated communication, public or community engagement specialists to support researchers Institute grants to promote public engagement with science
Build a culture of public engagement	Integrate public engagement in institutional and funding mandates and strategy Create policies and guidelines for public engagement at institutional-level Sensitise staff, researchers and students about the value of public engagement

## Conclusions

This survey is our first attempt at understanding the perspectives of a small subset of Indian researchers on public engagement to inform our strategy for enabling communication of and engagement with science in India. We acknowledge some degree of response bias in this survey. India Alliance actively promotes public engagement with science and encourages the participation of its grantees; ergo, the responders may be expected to have a relatively better understanding of public engagement in comparison to other research communities in the country. The survey may also be susceptible to some degree of self-reporting bias specially for questions requiring respondents to assess their skills and/or needs. Further, it is possible that grantees of India Alliance who are interested in public engagement responded to the survey and those not interested did not respond. Therefore, further studies would require more in-depth analysis and strategies to control reporting biases.

Lastly, this survey is anchored in the international public engagement survey run by Wellcome in 2016 that gathered views on public and community engagement with research across Africa and India ^13^, including India Alliance grantees. Through the present survey, we wanted to build on the previous findings from India and look more closely at the perspectives around public engagement with science in India. Additionally, in the absence of a framework for public engagement in India, the public engagement programmes of India Alliance have been informed by frameworks of Wellcome in the UK, Asia and Africa. Therefore, the approach in this survey is informed largely by the practices of the UK research ecosystem; however, it will be useful to take into account the practices and developments of multiple research ecosystems, including Asia-centric perspectives, in follow-up studies to design approaches to strengthen public engagement in India.

In the future, such studies can be expanded to include voices of researchers from diverse fields, public and community engagement practitioners, policymakers and funders. Further, the survey observations can be augmented with discussions and listening sessions with communities to better interpret the findings of the survey in light of community-specific contexts. Complemented by enquiries into the public's perception of science and its access to and engagement with scientific research, these studies will hopefully inform and develop thinking and practice around public engagement in the country. These will also provide the much-needed evidence base for formulating and implementing national policies and strategies as outlined in SSR ^3^ and STIP2020 ^4^ for promoting public engagement with science in India.

## Data availability

### Underlying data

Zenodo: Survey Data: Public Engagement with Science: A Survey for India Alliance Grantees, https://doi.org/10.5281/zenodo.6157335 ^19^.

This project contains de-identified responses of the researchers to the survey. Some key identifiers such as the name of the researcher, their host institution, and location have been removed. Additionally, identifiers have been removed from some of the comments in the open-ended questions of the survey. This is done to protect the respondents from any unintended consequences of their responses in the survey.

### Extended data

Zenodo: Survey Questionnaire: Public Engagement with Science: A Survey for India Alliance Grantees, https://doi.org/10.5281/zenodo.6157388 ^20^.

Zenodo: Data Graphs | Public Engagement with Science: A Survey for India Alliance Grantees [Data set]. https://doi.org/10.5281/zenodo.6376131 ^21^

Data are available under the terms of the Creative Commons Attribution 4.0 International license (CC-BY 4.0).
