# Supplementary material for: A survey to gather perspectives of DBT/Wellcome Trust India Alliance-funded researchers on public engagement with science
Source: Wellcome Open Res. 2022 Apr 8;6:269. Originally published 2021 Oct 14. [Version 2] doi: 10.12688/wellcomeopenres.17120.2 (PMC9021671; doi:10.12688/wellcomeopenres.17120.2)
Supplement: Peer review history

# Peer review: Reviewer response for version 2

**DOI:** 10.21956/wellcomeopenres.19466.r49888

**Published:** 2022-04-11

**Authors:** Marina Joubert

**Affiliations:** 1 Centre for Research on Evaluation, Science and Technology (CREST), Stellenbosch University, Stellenbosch, South Africa

I am satisfied with the amendments made by the authors. The updated version reads well and it is a welcome addition to research in the field.

Is the work clearly and accurately presented and does it cite the current literature?

Yes

If applicable, is the statistical analysis and its interpretation appropriate?

I cannot comment. A qualified statistician is required.

Are all the source data underlying the results available to ensure full reproducibility?

Yes

Is the study design appropriate and is the work technically sound?

Yes

Are the conclusions drawn adequately supported by the results?

Yes

Are sufficient details of methods and analysis provided to allow replication by others?

Yes

Reviewer Expertise:

Public communication of science.

I confirm that I have read this submission and believe that I have an appropriate level of expertise to confirm that it is of an acceptable scientific standard.

# Peer review: Reviewer response for version 2

**DOI:** 10.21956/wellcomeopenres.19466.r49889

**Published:** 2022-04-11

**Authors:** Mary Chambers, Han Dong Thai Tran

**Affiliations:** 1 Oxford University Clinical Research Unit, Ho Chi Minh City, Vietnam; 2 Tropical Medicine, University of Oxford, Oxford, UK

The authors have responded to our review and comments thoroughly. The revised paper is clearer and has better structure. Of particular importance is the fact they give more detailed recommendations for strengthening the PCE ecosystem in India. We hope that their work is a significant step in supporting this.

Is the work clearly and accurately presented and does it cite the current literature?

Yes

If applicable, is the statistical analysis and its interpretation appropriate?

Partly

Are all the source data underlying the results available to ensure full reproducibility?

Partly

Is the study design appropriate and is the work technically sound?

Yes

Are the conclusions drawn adequately supported by the results?

Yes

Are sufficient details of methods and analysis provided to allow replication by others?

Yes

Reviewer Expertise:

Public and community engagement

We confirm that we have read this submission and believe that we have an appropriate level of expertise to confirm that it is of an acceptable scientific standard.

# Peer review: Reviewer response for version 1

**DOI:** 10.21956/wellcomeopenres.18911.r46496

**Published:** 2021-11-05

**Authors:** Mary Chambers, Han Dong Thai Tran

**Affiliations:** 1 Oxford University Clinical Research Unit, Ho Chi Minh City, Vietnam; 2 Tropical Medicine, University of Oxford, Oxford, UK

**Review by Chambers and Tran**

 There is an increasing expectation that researchers engage with public and research communities, and there is increasing motivation from researchers to do so. However, the reality is that many researchers are not involved in public engagement. In order to better support them in this role, it is important to understand the motivators and challenges they face. The research conducted by Iqbal and Kar is a step forwards to understanding the engagement situation for researchers in India, and is important in outlining possible steps to support them. In this paper, the authors present findings from a survey of the perspectives of participating researchers at research institutes and higher education establishments in India about their practice of public engagement (PE) in science and research. The survey builds on a global survey conducted in 2016 by Wellcome. Consistency in questions and approach is helpful for a comparison of the local climate in India with global trends, although this isn’t discussed in the paper.

 The study captures the understanding of, participation in, and attitude of participants towards PE with research (as indicated in the abstract). The questionnaire was designed to capture respondents’ opinions. Data analysis is basic but sufficient to draw conclusions. Results from parallel questions were compared, showing consistency in responses, thus emphasizing the key findings. We laud the authors for the focus of their analysis on drawing out recommendations for steps necessary to further strengthen the research ecosystem and support Indian researchers to be better able to conduct PE. For these reasons we would strongly like to see this study indexed with the following amendments:  

**Our comments and recommendations**

**METHODS**

- A summary of the themes explored in the questionnaire could be included. Assume most readers won’t look at the appendices. We were not able to access the questionnaire - it seems the excel file is corrupted.
- Give demographic data about a wider sample of researchers in India. Who didn’t respond?
- Discuss the representativeness of the respondents much earlier in the paper.
- We wondered if there was bias because the respondents had received IA funding and therefore might feel obliged to report favorably. How was the survey kept anonymous when sent by email?
- Clarify what does “working as an independent researcher” mean regarding the stage in academia for a researcher. This will help with understanding the recommendation to incentivize public engagement (Please see specific comments for Discussion).

**RESULTS**

- Although it was good to see so much analysis had been done, we felt that there were too many figures and the quality of the figures was low. We suggest that the authors tabulate much of the data and reduce to 3-4 figures.
- We also felt there were too many quotes included. Summarise the responses and choose 1-2 key quotes to illustrate each theme.
- Be more consistent indicating throughout the Results section what types of questions were asked, e.g. Likert scale or Open-ended, and when respondents gave answers outside the list of options provided.
- To make the main answers more visible the top common (e.g. adding a summary table of answers about challenges, enablers, training skills needed.) At the moment the key findings are lost in the text and graphs and hard to recall.

*Section “Participation in public engagement”:*

- PE as the responsibility of researchers: It could be useful to explore the perspectives of people who disagreed or stayed neutral/don't know; why they think so? Any barriers, challenges indicated in the other questions linked to that? These could be included in the Discussion to suggest how future support could try to change that.
- It's worth exploring reasons for those who got the opportunity to do PE but did not participate (can this be explained from their responses for other questions?). Are these people the same as those who disagreed/stayed neutral with "PE is the responsibility of researchers?" Data of these two questions can be analyzed together to explore deeper into respondents' perspectives.
- Any of the people who responded "did not participate in any PE in last 2 years" indicated they wanted to spend more time. That could be interesting and correlates with challenges they've had to not participate.
- India Alliance fellows shared their views on the PE funding program of India. Did they have any specific inputs related to recommendations for IA PE programs?

**DISCUSSION**

- We would like to see more discussion about how the survey results measure the impact of the IA support and training for public engagement. Is IA doing something right that enables their fellows and recipients to conduct PE in a way that other organisations are not doing, and so non-IA researchers don’t receive this support? We feel that teasing this out is important to understand what is already working in the Indian context.

- We also feel that the discussion would be strengthened with a paragraph as to how these results compare with findings from the 2016 global survey.
- Finally, adding a box/summary list with bullet point recommendations will help the reader get those important take-home messages.

Is the work clearly and accurately presented and does it cite the current literature?

Yes

If applicable, is the statistical analysis and its interpretation appropriate?

Partly

Are all the source data underlying the results available to ensure full reproducibility?

Partly

Is the study design appropriate and is the work technically sound?

Yes

Are the conclusions drawn adequately supported by the results?

Yes

Are sufficient details of methods and analysis provided to allow replication by others?

Yes

Reviewer Expertise:

Public and community engagement

We confirm that we have read this submission and believe that we have an appropriate level of expertise to confirm that it is of an acceptable scientific standard, however we have significant reservations, as outlined above.

# Response

**Published:** 2022-03-22

**Authors:** Sarah Hyder Iqbal

The authors would like to thank the reviewers for their valuable feedback. Responses to their questions/comments are shared below:

**METHODS**

- **A summary of the themes explored in the questionnaire could be included. Assume most readers won’t look at the appendices. We were not able to access the questionnaire - it seems the excel file is corrupted.**

The survey explores three major themes: understanding of, participation in, and attitude towards public engagement with science; enablers, challenges, and barriers to public engagement; and specific needs (training/capacity building, funding, etc.) for public engagement. This has been included in the introduction of the manuscript. However, for further clarity a note on the themes has been added to the methods section.

- **Give demographic data about a wider sample of researchers in India. Who didn’t respond?**

Since some of the researchers chose to be anonymous in the survey, it is difficult to conclusively comment on the demography of the group who did not respond. However, from the data available, it can be said that a majority of the non-responders were intermediate fellowship awardees (>4 years post-PhD) of India Alliance. We have now included this under the methods sections.

- **Discuss the representativeness of the respondents much earlier in the paper.**

Based on the time spent post-PhD, the respondents have been grouped into early-career, mid-career, and senior researchers. This distribution has now been included under the methods section.

- **We wondered if there was bias because the respondents had received IA funding and therefore might feel obliged to report favorably. How was the survey kept anonymous when sent by email?**

We acknowledge some degree of bias in the survey which has been discussed in the conclusion section of the manuscript; we, therefore, propose follow-up studies with wider participation and strategies to address such biases.

 A link to the survey was shared by email (addresses were on BCC). Participants could remain anonymous by not sharing their personal information, which were non-mandatory fields on the survey. Before data submission, care was taken to anonymise the data per the requirements of WOR.  

- **Clarify what does “working as an independent researcher” mean regarding the stage in academia for a researcher. This will help with understanding the recommendation to incentivize public engagement (Please see specific comments for Discussion).**

“Working as an independent researcher” refers to the time spent as a researcher post-PhD (independently managing their projects).

**RESULTS**

- **Although it was good to see so much analysis had been done, we felt that there were too many figures and the quality of the figures was low. We suggest that the authors tabulate much of the data and reduce to 3-4 figures.**

As suggested, we have removed figures for sections wherein data could be represented sufficiently well through text. As suggested later, we have also added a summary table. We consider retaining the rest of the figures which aids in better representation of the data.  

- **We also felt there were too many quotes included. Summarise the responses and choose 1-2 key quotes to illustrate each theme.**

Changes were made in the text as suggested in the new version of the manuscript.

- **Be more consistent indicating throughout the Results section what types of questions were asked, e.g. Likert scale or Open-ended, and when respondents gave answers outside the list of options provided.**

The text has been modified as suggested in the new version of the manuscript.

- **To make the main answers more visible the top common (e.g. adding a summary table of answers about challenges, enablers, training skills needed.) At the moment the key findings are lost in the text and graphs and hard to recall.**

We have now included a summary table in the results section in the new version. 

**Section “Participation in public engagement”:**

- **PE as the responsibility of researchers: It could be useful to explore the perspectives of people who disagreed or stayed neutral/don't know; why they think so? Any barriers, challenges indicated in the other questions linked to that? These could be included in the Discussion to suggest how future support could try to change that.**

No differences were observed for the people who disagreed or stayed neutral in terms of their interest or understanding of PE. 

- **It's worth exploring reasons for those who got the opportunity to do PE but did not participate (can this be explained from their responses for other questions?). Are these people the same as those who disagreed/stayed neutral with "PE is the responsibility of researchers?" Data of these two questions can be analyzed together to explore deeper into respondents' perspectives.**

These details have been added to the new version of the manuscript.

- **Any of the people who responded "did not participate in any PE in last 2 years" indicated they wanted to spend more time. That could be interesting and correlates with challenges they've had to not participate.**

These details have been added to the new version of the manuscript.

- **India Alliance fellows shared their views on the PE funding program of India. Did they have any specific inputs related to recommendations for IA PE programs?**

No specifics inputs were received for the PE funding program of India Alliance. 

**DISCUSSION**

- **We would like to see more discussion about how the survey results measure the impact of the IA support and training for public engagement. Is IA doing something right that enables their fellows and recipients to conduct PE in a way that other organisations are not doing, and so non-IA researchers don’t receive this support? We feel that teasing this out is important to understand what is already working in the Indian context.**

The survey reported here focused on understanding the needs of researchers funded by IA so that appropriate support can be provided to them to undertake public engagement activities as part of their fellowship or grant programme. The survey did not intend to evaluate IA’s current support for PE or draw a comparison with other organisations in the country but we recognise that this will be an important step in the process of developing an effective PE support programme for the grantees. 
**  **

- **We also feel that the discussion would be strengthened with a paragraph as to how these results compare with findings from the 2016 global survey.**

These details have been added to the new version of the manuscript. 

- **Finally, adding a box/summary list with bullet point recommendations will help the reader get those important take-home messages.**

A summary table has been added to the new version of the manuscript.

# Peer review: Reviewer response for version 1

**DOI:** 10.21956/wellcomeopenres.18911.r46497

**Published:** 2021-11-04

**Authors:** Marina Joubert

**Affiliations:** 1 Centre for Research on Evaluation, Science and Technology (CREST), Stellenbosch University, Stellenbosch, South Africa

This manuscript reports on a survey of grant-holders of the India Alliance to determine their views on public engagement with science. Since scientists themselves (for example, researchers funded through agencies and charities) play a key role in science engagement, it is important to understand their views, as well as the benefits and risks they perceive when engaging with public audiences. That is also why a considerable amount of research has lately been focused on scientists’ objectives and views regarding engagement. However, as the authors note, this study may be the first of its kind in India (since much of this kind of research has focused on scientists in developed countries).

 In general, it is worth pointing out that this survey builds on other similar studies (as also acknowledged by the authors). In particular, the authors say that the questions were modeled on a similar survey carried out by Wellcome in 2016 to gather the views of researchers on public engagement in Asia and Africa. This led to the expectation that there may have been a bit more (in the discussion section) about how the findings of this survey compare with the 2016 survey. While the authors make ample reference to relevant earlier literature, the current research is not supported by a theoretical lens but takes a very practical approach. The authors note "the absence of a framework for public engagement in India...", something that could be highlighted for future research.

 The overall findings (that grantees are largely motivated to engage with the public about science or their research but lack professional recognition and incentives, training, and structural support to undertake public engagement activities) are largely in line with what other studies have shown. In this regard, I was hoping to find something in the study – both in terms of its design and its findings – that would shed light on the unique context in India. In other words, I would have loved to find out more about incentives and impediments that maybe influenced the politics, policies, and cultural conditions in India, that could shape scientists’ views about engagement.

 The introduction is well written and provides a good backdrop to the study, both globally and within the local (Indian) context. It is clear the India Alliance needs to gain a better understanding of how their grant-holders respond to demands for public engagement, and this is linked to the objectives of the current study.

 The authors note that the full survey questionnaire is available as extended data, but I could not download the files due to a persistent error message when I tried to access it via the link provided. This is possibly due to a problem on my side, and I’m still trying to access the full survey design. In any case, it may be good to add (to the methodology section) the main topics addressed in the survey, such as understanding, perceptions, participation, etc.

 The findings are presented clearly and visually in the results section, and the illustrative quotes add further insight to the findings. I also found the discussion section useful and agreed with the way the authors reflected on their findings. It is also useful that the authors conclude with a set of recommendations. I have a few minor suggestions regarding the methods section:

1. Add a date (exactly when the survey was issued) at the start of the methodology, as well as the information about how the grantees were contacted and reminded.
2. Did India Alliance have only 243 grantees at the time? Make it clear.
3. You mention “243 Indian researchers in receipt of India Alliance fellowships or grants”, does this mean that you excluded grant-holders of other nationalities (were there any?)
4. You say that 137 responded, add a response rate (%).
5. I’m not sure what this means: “…the data was automatically mapped in Microsoft Excel” – explain, please.

Concern regarding ethics:

 The authors note that “The study did not undergo a formal ethical clearance process as the primary objective of the study was to gather insights of grantees to inform India Alliance’s public engagement support mechanisms.” I’m not sure that this is a good reason to skip ethical clearance. I guess this depends on the policy at India Alliance. In the institution where I work, researchers would not be allowed to gather this kind of data, let alone publish it, without prior ethical clearance. Perhaps the authors can add a note to better explain why ethical clearance was not obtained. Possibly it was not necessary (according to the policies of India Alliance) given that the responses were anonymised?

Is the work clearly and accurately presented and does it cite the current literature?

Yes

If applicable, is the statistical analysis and its interpretation appropriate?

I cannot comment. A qualified statistician is required.

Are all the source data underlying the results available to ensure full reproducibility?

Yes

Is the study design appropriate and is the work technically sound?

Yes

Are the conclusions drawn adequately supported by the results?

Yes

Are sufficient details of methods and analysis provided to allow replication by others?

Yes

Reviewer Expertise:

Public communication of science.

I confirm that I have read this submission and believe that I have an appropriate level of expertise to confirm that it is of an acceptable scientific standard, however I have significant reservations, as outlined above.

# Response

**Published:** 2022-03-22

**Authors:** Sarah Hyder Iqbal

The authors would like to thank Dr. Joubert for her constructive and helpful feedback. Responses to her comments/questions has been shared below: 

**1. The information about how the grantees were contacted and reminded.**

 The survey was created on Microsoft Forms and a link was generated which was shared by email with the India Alliance grantees on 27 November 2020. The data was collected from 27 November 2020. Reminders were also sent by email. This information has been updated in the manuscript.

**2. Did India Alliance have only 243 grantees at the time? Make it clear.**

 The survey was sent to the active and approved grantees of India Alliance and not the ones with terminated or completed grants. We have now added this information to the manuscript.

**3. You mention “243 Indian researchers in receipt of India Alliance fellowships or grants”, does this mean that you excluded grant-holders of other nationalities (were there any?)**

 The cohort of grantees surveyed did not include any grant-holders of foreign nationalities; we did not exclude any grant-holder based on their nationality. (Although the India Alliance programs do not have any nationality restrictions, the grantees are to carry out their work at a host institution based in India.)

**4. You say that 137 responded, add a response rate (%).**

 The response rate of the survey was 56.4%. We have now added this to the manuscript.

**5. I’m not sure what this means: “…the data was automatically mapped in Microsoft Excel” – explain, please.**

 As the survey was shared in the form of a Microsoft Form, the responses from the survey could be downloaded in Microsoft Excel format. This data was checked, cleaned, and anonymised for submission. Using Microsoft Excel, the data was also plotted to generate graphs for submission with the manuscript.

**Concern regarding ethics:**

**The authors note that “The study did not undergo a formal ethical clearance process as the primary objective of the study was to gather insights of grantees to inform India Alliance’s public engagement support mechanisms.” I’m not sure that this is a good reason to skip ethical clearance. I guess this depends on the policy at India Alliance. In the institution where I work, researchers would not be allowed to gather this kind of data, let alone publish it, without prior ethical clearance. Perhaps the authors can add a note to better explain why ethical clearance was not obtained. Possibly it was not necessary (according to the policies of India Alliance) given that the responses were anonymised?**

 This has been addressed in the methods section.
